# Supplementary material for: A Likelihood Approach for Real-Time Calibration of Stochastic Compartmental Epidemic Models
Source: PLoS Comput Biol. 2017 Jan 17;13(1):e1005257. doi: 10.1371/journal.pcbi.1005257 (PMC5240920; doi:10.1371/journal.pcbi.1005257)
Supplement: S1 File — (TAR.GZ) [file pcbi.1005257.s014.tar.gz › HSPH_Online-SI-Revision/MSS10/n10-mild/n10-mild_NEW2_table4.pdf]

1	newly 3 weeks spec	153.	84.65	{12., 152.}	50.5294	0.484552	newly 3 weeks cumul	425.	317.95	{102., 544.}	39.1788	0.279154	newly 5 weeks spec	103.	54.59	{0., 125.}	56.7864	0.802245	newly 5 weeks cumul	653.	442.38	{118., 814.}	42.4472	0.324393	newly 46 weeks cumul	1470.	605.1	{120., 1102.}	58.8367	0.532343
2	newly 3 weeks spec	110.	56.77	{23., 84.}	48.6273	0.382272	newly 3 weeks cumul	464.	284.41	{153., 402.}	39.0927	0.250737	newly 5 weeks spec	57.	23.83	{3., 47.}	59.2807	0.58982	newly 5 weeks cumul	610.	346.05	{181., 485.}	43.359	0.28612	newly 51 weeks cumul	742.	389.73	{194., 589.}	47.6348	0.32444
3	newly 3 weeks spec	211.	100.09	{43., 154.}	52.564	0.404716	newly 3 weeks cumul	768.	518.77	{311., 697.}	32.8424	0.196443	newly 5 weeks spec	97.	37.16	{4., 73.}	62.0825	0.661934	newly 5 weeks cumul	973.	616.21	{342., 850.}	36.6917	0.227715	newly 54 weeks cumul	1157.	686.61	{342., 1040.}	40.656	0.264034
4	newly 3 weeks spec	113.	118.75	{88., 154.}	18.3628	0.0776447	newly 3 weeks cumul	452.	515.5	{410., 618.}	17.8274	0.0701596	newly 5 weeks spec	81.	56.86	{34., 81.}	33.037	0.196371	newly 5 weeks cumul	619.	655.71	{524., 806.}	13.4362	0.0559178	newly 49 weeks cumul	827.	770.82	{632., 944.}	14.2418	0.0678508
5	newly 3 weeks spec	93.	85.04	{55., 116.}	21.5269	0.106477	newly 3 weeks cumul	276.	274.82	{201., 339.}	15.8768	0.0714183	newly 5 weeks spec	92.	67.51	{42., 99.}	32.8804	0.18566	newly 5 weeks cumul	472.	419.06	{312., 529.}	18.0763	0.089422	newly 29 weeks cumul	1568.	804.28	{540., 1121.}	48.7066	0.309011
6	newly 3 weeks spec	108.	79.14	{17., 142.}	43.3333	0.310762	newly 3 weeks cumul	406.	329.8	{94., 565.}	38.734	0.240981	newly 5 weeks spec	80.	35.19	{1., 73.}	59.9625	0.68346	newly 5 weeks cumul	611.	418.36	{109., 701.}	40.9853	0.284292	newly 55 weeks cumul	829.	490.28	{116., 847.}	45.3993	0.338459
7	newly 3 weeks spec	89.	74.27	{20., 124.}	38.9775	0.265324	newly 3 weeks cumul	391.	348.14	{152., 528.}	30.7468	0.177395	newly 5 weeks spec	87.	30.91	{2., 66.}	64.6552	0.745625	newly 5 weeks cumul	552.	427.59	{162., 663.}	33.1178	0.212177	newly 51 weeks cumul	801.	485.74	{187., 786.}	42.427	0.298641
8	newly 3 weeks spec	197.	102.45	{43., 172.}	48.7056	0.349156	newly 3 weeks cumul	615.	440.83	{196., 632.}	32.5057	0.198617	newly 5 weeks spec	164.	48.27	{16., 86.}	70.5671	0.638075	newly 5 weeks cumul	920.	561.15	{248., 810.}	39.9946	0.258713	newly 46 weeks cumul	2034.	644.91	{284., 970.}	68.2935	0.541637
9	newly 3 weeks spec	116.	144.93	{107., 186.}	29.4569	0.108237	newly 3 weeks cumul	592.	621.31	{502., 735.}	12.6571	0.0530322	newly 5 weeks spec	85.	68.03	{44., 92.}	25.3059	0.133485	newly 5 weeks cumul	760.	789.59	{642., 928.}	13.0066	0.0550051	newly 50 weeks cumul	872.	920.97	{742., 1073.}	13.2603	0.054782
10	newly 3 weeks spec	91.	74.08	{30., 122.}	35.6264	0.206074	newly 3 weeks cumul	345.	323.74	{168., 484.}	27.4841	0.134068	newly 5 weeks spec	65.	40.3	{9., 75.}	48.8	0.43928	newly 5 weeks cumul	482.	415.43	{206., 631.}	29.5954	0.156353	newly 47 weeks cumul	1027.	497.57	{241., 743.}	51.5511	0.350967
11	newly 3 weeks spec	165.	29.07	{0., 72.}	82.503	1.34947	newly 3 weeks cumul	495.	178.5	{35., 323.}	64.4242	0.590118	newly 5 weeks spec	56.	12.45	{0., 44.}	82.5536	1.83745	newly 5 weeks cumul	648.	209.1	{35., 407.}	68.1265	0.66271	newly 52 weeks cumul	801.	241.21	{35., 555.}	71.427	0.730903
12	newly 3 weeks spec	53.	29.37	{4., 59.}	53.9434	0.498699	newly 3 weeks cumul	224.	197.68	{92., 312.}	34.7679	0.199565	newly 5 weeks spec	9.	8.5	{0., 24.}	97.3333	0.933316	newly 5 weeks cumul	264.	222.47	{100., 353.}	35.7992	0.213843	newly 54 weeks cumul	276.	231.21	{101., 361.}	36.7645	0.221461
13	newly 3 weeks spec	135.	104.73	{71., 149.}	26.5852	0.141433	newly 3 weeks cumul	528.	463.48	{361., 570.}	16.7879	0.0821598	newly 5 weeks spec	51.	50.29	{26., 73.}	29.9412	0.138883	newly 5 weeks cumul	676.	588.76	{455., 735.}	17.7278	0.0871176	newly 46 weeks cumul	774.	702.74	{522., 890.}	17.4367	0.0834753
14	newly 3 weeks spec	150.	111.06	{64., 166.}	33.7333	0.191441	newly 3 weeks cumul	578.	462.11	{314., 630.}	24.9913	0.131697	newly 5 weeks spec	106.	55.	{20., 91.}	51.1132	0.361199	newly 5 weeks cumul	857.	596.75	{419., 819.}	32.9907	0.185292	newly 47 weeks cumul	1338.	707.95	{455., 1132.}	47.6928	0.304872
15	newly 3 weeks spec	208.	121.71	{60., 188.}	41.8221	0.274366	newly 3 weeks cumul	656.	463.85	{264., 665.}	30.9527	0.189337	newly 5 weeks spec	192.	71.06	{25., 117.}	62.9896	0.498039	newly 5 weeks cumul	1065.	630.38	{330., 921.}	40.8094	0.260214	newly 49 weeks cumul	2326.	810.87	{373., 1216.}	65.1389	0.496443
16	newly 3 weeks spec	196.	105.02	{69., 143.}	46.4184	0.291305	newly 3 weeks cumul	619.	467.18	{350., 581.}	24.937	0.132702	newly 5 weeks spec	104.	47.77	{22., 75.}	54.0865	0.401437	newly 5 weeks cumul	881.	585.86	{427., 742.}	33.6118	0.187744	newly 50 weeks cumul	1128.	669.32	{483., 872.}	40.6631	0.238574
17	newly 3 weeks spec	103.	75.44	{0., 175.}	54.9515	0.599508	newly 3 weeks cumul	286.	264.41	{34., 539.}	53.7727	0.352591	newly 5 weeks spec	112.	47.24	{0., 114.}	61.875	0.991741	newly 5 weeks cumul	495.	372.52	{34., 752.}	48.9051	0.4005	newly 47 weeks cumul	2645.	556.64	{34., 1149.}	78.955	0.919584
18	newly 3 weeks spec	134.	111.14	{56., 179.}	31.1194	0.177191	newly 3 weeks cumul	498.	403.31	{268., 568.}	25.8414	0.141262	newly 5 weeks spec	79.	66.84	{24., 113.}	39.3671	0.272064	newly 5 weeks cumul	686.	557.61	{350., 822.}	26.2114	0.145803	newly 47 weeks cumul	921.	743.38	{424., 1064.}	26.949	0.156546
19	newly 3 weeks spec	74.	33.24	{4., 66.}	59.5405	0.596602	newly 3 weeks cumul	333.	213.84	{74., 345.}	40.4625	0.27618	newly 5 weeks spec	49.	7.34	{0., 20.}	85.0204	1.5157	newly 5 weeks cumul	449.	236.46	{98., 384.}	48.9889	0.356221	newly 50 weeks cumul	666.	244.01	{98., 395.}	63.6111	0.512119
20	newly 3 weeks spec	165.	86.03	{45., 128.}	48.2	0.340331	newly 3 weeks cumul	661.	412.63	{271., 555.}	37.5749	0.223894	newly 5 weeks spec	141.	34.74	{5., 65.}	75.3617	0.799382	newly 5 weeks cumul	964.	503.81	{320., 711.}	47.7376	0.304608	newly 51 weeks cumul	1374.	561.1	{327., 827.}	59.163	0.417339
21	newly 3 weeks spec	122.	71.65	{39., 106.}	41.582	0.261566	newly 3 weeks cumul	466.	351.96	{232., 445.}	26.9957	0.147605	newly 5 weeks spec	62.	30.16	{7., 56.}	53.9355	0.453701	newly 5 weeks cumul	595.	428.19	{272., 557.}	29.6017	0.165172	newly 51 weeks cumul	738.	469.12	{276., 640.}	36.439	0.21526
22	newly 3 weeks spec	145.	71.28	{18., 113.}	53.2138	0.407193	newly 3 weeks cumul	458.	314.89	{123., 502.}	36.8319	0.231904	newly 5 weeks spec	86.	35.21	{2., 73.}	61.4302	0.642981	newly 5 weeks cumul	631.	400.47	{136., 617.}	40.0808	0.264272	newly 47 weeks cumul	1216.	473.13	{159., 754.}	61.1637	0.471591
23	newly 3 weeks spec	51.	61.21	{16., 105.}	55.0392	0.276302	newly 3 weeks cumul	384.	303.66	{120., 460.}	32.4896	0.205442	newly 5 weeks spec	14.	23.55	{0., 48.}	115.786	0.588055	newly 5 weeks cumul	436.	363.7	{142., 561.}	33.2202	0.202822	newly 52 weeks cumul	442.	403.83	{154., 651.}	34.7353	0.199424
24	newly 3 weeks spec	109.	64.68	{5., 123.}	53.211	0.520222	newly 3 weeks cumul	313.	263.06	{67., 471.}	42.3323	0.262108	newly 5 weeks spec	56.	30.32	{0., 66.}	61.9286	0.894521	newly 5 weeks cumul	432.	339.14	{71., 634.}	44.0185	0.297465	newly 48 weeks cumul	566.	409.	{71., 792.}	46.1237	0.341056
25	newly 3 weeks spec	134.	154.15	{66., 253.}	42.7239	0.184941	newly 3 weeks cumul	550.	678.77	{353., 983.}	41.7364	0.166612	newly 5 weeks spec	58.	71.	{24., 127.}	56.	0.248974	newly 5 weeks cumul	716.	854.78	{461., 1238.}	37.5894	0.151475	newly 55 weeks cumul	798.	982.37	{578., 1407.}	38.0288	0.147082
26	newly 3 weeks spec	59.	44.61	{3., 85.}	51.5424	0.500909	newly 3 weeks cumul	261.	216.25	{48., 356.}	38.9464	0.271958	newly 5 weeks spec	33.	15.6	{0., 39.}	66.7273	1.04484	newly 5 weeks cumul	353.	257.41	{48., 461.}	40.2351	0.311008	newly 46 weeks cumul	475.	280.49	{48., 497.}	45.8211	0.383308
27	newly 3 weeks spec	87.	80.49	{47., 115.}	25.3448	0.128821	newly 3 weeks cumul	397.	379.04	{269., 479.}	17.1537	0.0803644	newly 5 weeks spec	24.	36.79	{15., 57.}	68.5417	0.232237	newly 5 weeks cumul	477.	470.37	{315., 595.}	16.6646	0.0758547	newly 48 weeks cumul	499.	536.71	{352., 676.}	18.8156	0.0793658
28	newly 3 weeks spec	95.	58.75	{15., 101.}	44.7474	0.344699	newly 3 weeks cumul	273.	220.03	{93., 339.}	33.5568	0.209265	newly 5 weeks spec	72.	39.4	{6., 73.}	51.0833	0.496112	newly 5 weeks cumul	428.	307.06	{114., 476.}	35.8645	0.242273	newly 26 weeks cumul	1195.	451.34	{131., 765.}	62.231	0.515058
29	newly 3 weeks spec	140.	66.27	{32., 109.}	52.8643	0.404554	newly 3 weeks cumul	520.	414.88	{278., 590.}	26.6731	0.156233	newly 5 weeks spec	86.	17.82	{0., 40.}	79.2791	1.04695	newly 5 weeks cumul	706.	465.21	{287., 672.}	35.1487	0.220758	newly 49 weeks cumul	1008.	481.5	{289., 713.}	52.2321	0.358803
30	newly 3 weeks spec	99.	66.04	{27., 101.}	36.9293	0.26892	newly 3 weeks cumul	453.	339.45	{192., 461.}	27.9095	0.166801	newly 5 weeks spec	51.	27.01	{4., 56.}	55.7451	0.543893	newly 5 weeks cumul	566.	408.34	{227., 597.}	30.4594	0.186353	newly 48 weeks cumul	783.	446.53	{247., 673.}	43.0587	0.28342
31	newly 3 weeks spec	92.	100.65	{45., 161.}	38.25	0.190658	newly 3 weeks cumul	415.	398.91	{212., 614.}	30.1614	0.162792	newly 5 weeks spec	49.	59.59	{22., 103.}	53.2041	0.257587	newly 5 weeks cumul	552.	538.78	{277., 824.}	29.0978	0.156599	newly 55 weeks cumul	668.	688.36	{392., 956.}	28.9251	0.150849
32	newly 3 weeks spec	124.	62.97	{20., 102.}	49.7016	0.385625	newly 3 weeks cumul	386.	244.04	{120., 378.}	37.886	0.254045	newly 5 weeks spec	137.	35.25	{6., 76.}	74.2701	0.810498	newly 5 weeks cumul	648.	326.7	{135., 514.}	49.5833	0.353349	newly 28 weeks cumul	1971.	443.48	{172., 829.}	77.4997	0.722416
33	newly 3 weeks spec	96.	118.21	{79., 159.}	30.8646	0.114671	newly 3 weeks cumul	537.	515.7	{410., 626.}	12.6741	0.0581827	newly 5 weeks spec	49.	55.42	{30., 79.}	31.102	0.125978	newly 5 weeks cumul	666.	650.26	{500., 804.}	13.4925	0.0610096	newly 49 weeks cumul	775.	762.69	{573., 962.}	14.7006	0.0662716
34	newly 3 weeks spec	217.	74.94	{23., 146.}	65.4654	0.583758	newly 3 weeks cumul	685.	390.7	{162., 618.}	43.7051	0.310035	newly 5 weeks spec	140.	27.03	{0., 65.}	80.6929	1.12082	newly 5 weeks cumul	982.	461.31	{175., 760.}	53.0234	0.395308	newly 49 weeks cumul	1514.	506.19	{177., 821.}	66.5661	0.545742
35	newly 3 weeks spec	115.	64.6	{31., 102.}	44.8348	0.306574	newly 3 weeks cumul	444.	317.78	{218., 404.}	29.4369	0.165321	newly 5 weeks spec	59.	27.6	{6., 52.}	55.1864	0.481679	newly 5 weeks cumul	616.	387.32	{252., 499.}	37.461	0.222034	newly 49 weeks cumul	837.	441.34	{266., 614.}	47.3381	0.303015
36	newly 3 weeks spec	124.	35.74	{8., 63.}	71.1774	0.670676	newly 3 weeks cumul	489.	267.34	{125., 421.}	45.6115	0.306459	newly 5 weeks spec	43.	10.3	{0., 28.}	76.3256	1.2215	newly 5 weeks cumul	588.	296.06	{140., 466.}	49.7041	0.34266	newly 51 weeks cumul	620.	305.09	{140., 482.}	50.8435	0.353742
37	newly 3 weeks spec	61.	45.74	{0., 105.}	59.3115	0.591741	newly 3 weeks cumul	281.	236.65	{40., 415.}	46.2242	0.331742	newly 5 weeks spec	9.	16.53	{0., 40.}	170.778	0.962481	newly 5 weeks cumul	307.	282.35	{40., 540.}	50.5049	0.341112	newly 54 weeks cumul	480.	310.83	{40., 605.}	49.4229	0.419972
38	newly 3 weeks spec	169.	86.06	{30., 144.}	49.2781	0.408887	newly 3 weeks cumul	582.	441.58	{260., 607.}	26.5876	0.153872	newly 5 weeks spec	127.	35.74	{1., 72.}	71.8583	0.858007	newly 5 weeks cumul	845.	533.08	{308., 743.}	37.1669	0.228575	newly 50 weeks cumul	1549.	588.85	{330., 830.}	61.9852	0.450319
39	newly 3 weeks spec	23.	30.35	{5., 64.}	76.3043	0.372575	newly 3 weeks cumul	145.	200.59	{85., 314.}	60.1862	0.221341	newly 5 weeks spec	14.	10.04	{0., 29.}	80.5714	0.971489	newly 5 weeks cumul	176.	226.67	{89., 376.}	54.5398	0.216033	newly 51 weeks cumul	182.	239.59	{89., 411.}	57.3901	0.224029
40	newly 3 weeks spec	69.	58.63	{33., 89.}	28.6522	0.153876	newly 3 weeks cumul	223.	193.54	{130., 260.}	21.426	0.1105	newly 5 weeks spec	71.	49.78	{19., 80.}	37.662	0.239044	newly 5 weeks cumul	369.	297.33	{191., 410.}	24.5718	0.134963	newly 24 weeks cumul	1107.	556.5	{295., 861.}	49.729	0.332566
41	newly 3 weeks spec	7.	26.95	{0., 64.}	337.857	0.874758	newly 3 weeks cumul	98.	171.74	{18., 369.}	118.571	0.444017	newly 5 weeks spec	0.1	8.3	{0., 28.}	8290.	1.09119	newly 5 weeks cumul	102.	194.7	{18., 425.}	133.961	0.47208	newly 55 weeks cumul	102.	205.6	{18., 443.}	144.569	0.487256
42	newly 3 weeks spec	113.	95.84	{43., 154.}	33.7522	0.195605	newly 3 weeks cumul	500.	403.01	{229., 566.}	26.738	0.153721	newly 5 weeks spec	90.	49.36	{16., 89.}	46.9778	0.386416	newly 5 weeks cumul	679.	521.69	{282., 748.}	28.8999	0.17214	newly 47 weeks cumul	908.	628.88	{306., 943.}	34.0507	0.212849
43	newly 3 weeks spec	16.	53.9	{8., 91.}	252.875	0.560765	newly 3 weeks cumul	172.	350.29	{176., 532.}	107.692	0.299347	newly 5 weeks spec	1.	12.96	{0., 33.}	1234.	1.02119	newly 5 weeks cumul	187.	390.13	{183., 583.}	113.364	0.311605	newly 53 weeks cumul	187.	402.97	{189., 606.}	120.048	0.323063
44	newly 3 weeks spec	169.	99.49	{54., 146.}	41.7101	0.269263	newly 3 weeks cumul	606.	436.1	{263., 589.}	29.5083	0.170352	newly 5 weeks spec	127.	45.6	{16., 79.}	64.0945	0.552201	newly 5 weeks cumul	896.	549.1	{331., 737.}	39.0201	0.236658	newly 49 weeks cumul	1535.	619.75	{348., 865.}	59.6254	0.420347
45	newly 3 weeks spec	53.	41.93	{12., 75.}	41.4528	0.26473	newly 3 weeks cumul	403.	310.29	{138., 467.}	31.4814	0.185283	newly 5 weeks spec	9.	10.75	{0., 27.}	103.444	0.746222	newly 5 weeks cumul	435.	342.16	{174., 518.}	31.3517	0.182751	newly 56 weeks cumul	452.	353.55	{175., 547.}	32.7898	0.19159
46	newly 3 weeks spec	126.	124.04	{73., 183.}	25.2857	0.118057	newly 3 weeks cumul	521.	529.14	{373., 694.}	19.9463	0.0886551	newly 5 weeks spec	38.	58.96	{24., 95.}	72.5263	0.239485	newly 5 weeks cumul	617.	676.	{471., 907.}	24.0194	0.0994635	newly 53 weeks cumul	658.	791.74	{525., 1105.}	31.4438	0.121262
47	newly 3 weeks spec	195.	100.72	{50., 149.}	48.4205	0.328119	newly 3 weeks cumul	736.	548.32	{355., 759.}	27.7663	0.155581	newly 5 weeks spec	94.	32.58	{5., 61.}	65.5532	0.643559	newly 5 weeks cumul	971.	640.14	{402., 891.}	34.62	0.203761	newly 53 weeks cumul	1232.	683.28	{405., 938.}	44.539	0.278803
48	newly 3 weeks spec	116.	68.43	{21., 121.}	45.5086	0.38933	newly 3 weeks cumul	481.	355.27	{180., 545.}	34.2266	0.209852	newly 5 weeks spec	50.	25.96	{0., 63.}	62.76	0.849027	newly 5 weeks cumul	630.	422.89	{191., 655.}	37.8143	0.24555	newly 50 weeks cumul	758.	465.16	{192., 732.}	41.5409	0.281033
49	newly 3 weeks spec	138.	108.78	{36., 174.}	33.2754	0.200503	newly 3 weeks cumul	440.	412.6	{225., 634.}	28.6727	0.14336	newly 5 weeks spec	140.	67.54	{12., 116.}	52.1429	0.436282	newly 5 weeks cumul	714.	568.31	{278., 847.}	29.4748	0.169853	newly 46 weeks cumul	1855.	762.74	{335., 1109.}	58.8819	0.422751
50	newly 3 weeks spec	69.	45.77	{4., 79.}	44.7681	0.447137	newly 3 weeks cumul	243.	202.84	{62., 359.}	35.4074	0.231412	newly 5 weeks spec	43.	19.82	{0., 43.}	59.8605	0.71277	newly 5 weeks cumul	357.	253.57	{62., 436.}	38.6751	0.280269	newly 46 weeks cumul	518.	291.29	{62., 518.}	47.1293	0.369937
